# Cellular senescence as a potential mediator of COVID‐19 severity in the elderly

**DOI:** 10.1111/acel.13237

**Published:** 2020-09-21

**Authors:** Jamil Nehme, Michela Borghesan, Sebastian Mackedenski, Thomas G. Bird, Marco Demaria

**Affiliations:** ^1^ European Research Institute for the Biology of Ageing (ERIBA) University Medical Center Groningen (UMCG) University of Groningen (RUG Groningen NL The Netherlands; ^2^ Doctoral School of Science and Technology Lebanese University Beirut Lebanon; ^3^ Cancer Research UK Beatson Institute Glasgow UK; ^4^ Institute of Cancer Sciences University of Glasgow Glasgow UK; ^5^ MRC Centre for Inflammation Research The Queen's Medical Research Institute University of Edinburgh Edinburgh UK

## Abstract

SARS‐CoV‐2 is a novel betacoronavirus which infects the lower respiratory tract and can cause coronavirus disease 2019 (COVID‐19), a complex respiratory distress syndrome. Epidemiological data show that COVID‐19 has a rising mortality particularly in individuals with advanced age. Identifying a functional association between SARS‐CoV‐2 infection and the process of biological aging may provide a tractable avenue for therapy to prevent acute and long‐term disease. Here, we discuss how cellular senescence—a state of stable growth arrest characterized by pro‐inflammatory and pro‐disease functions—can hypothetically be a contributor to COVID‐19 pathogenesis, and a potential pharmaceutical target to alleviate disease severity. First, we define why older COVID‐19 patients are more likely to accumulate high levels of cellular senescence. Second, we describe how senescent cells can contribute to an uncontrolled SARS‐CoV‐2‐mediated cytokine storm and an excessive inflammatory reaction during the early phase of the disease. Third, we discuss the various mechanisms by which senescent cells promote tissue damage leading to lung failure and multi‐tissue dysfunctions. Fourth, we argue that a high senescence burst might negatively impact on vaccine efficacy. Measuring the burst of cellular senescence could hypothetically serve as a predictor of COVID‐19 severity, and targeting senescence‐associated mechanisms prior and after SARS‐CoV‐2 infection might have the potential to limit a number of severe damages and to improve the efficacy of vaccinations.

## Graphical abstract

Senescence accumulation as a result of multiple factors can increase the risk of developing severe complications upon SARS‐CoV‐2 infection and cause poor vaccine response. Senescence biomarkers can serve to evaluate the risk of developing severe COVID‐19 and senotherapeutics as a good strategy to limit detrimental long‐term health consequences.

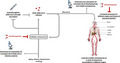

## 1 INTRODUCTION

Coronavirus disease 2019 (COVID‐19) represents a complex respiratory distress syndrome which can evolve into multi‐organ failure caused by the betacoronavirus SARS‐CoV‐2. Epidemiological data shows that COVID‐19 has a rising mortality particularly in males with advanced age. In United States, Europe, and China, individuals over 60 years of age with underlying conditions such as hypertension, diabetes, cardiovascular or respiratory disease, and cancer are considered to be more at risk to develop severe symptoms (Chen et al., 2020; Control, E.C.f.D.P.a.,; Garg et al., 2020; Huang et al., 2020; Liang et al., 2020; WHO; Zhou et al., 2020). Whether there is a functional association between SARS‐CoV‐2 infection and biological aging remains an open question.

Aging is a physiological decline of organismal functions involving numerous components. A major age‐associated hallmark is the accumulation of cells that undergo senescence—a state of stable growth arrest characterized by hypersecretory functions. The hypersecretion is collectively defined as SASP (senescence‐associated secretory phenotype) and includes various tissue remodeling (e.g., TGF‐β and MMPs) and immune‐related (e.g., IL‐6, IL‐8, and IFNs) factors. Via tissue remodeling SASP factors, senescent cells positively regulate tissue regeneration and repair, and help to maintain tissue homeostasis. Via immune‐related SASP factors, senescent cells promote immunosurveillance, particularly in the context of tumorigenesis where they recruit T cells and NK cells (Prata et al., 2018). In optimal conditions and in young organisms, senescent cells are only transiently present and are timely removed by either immune‐mediated mechanisms or by transitioning to other states, mainly apoptosis (Childs et al., 2014). In contrast, during aging and chronic diseases, these events do not function properly, and senescent cells persist (Munoz‐Espin & Serrano, 2014). Moreover, aged cells become more susceptible to damage, and more likely to undergo senescence (Bermúdez‐Cruz & F.A.L.‐R.a.R.M., 2019). These events contribute to a steady accumulation of senescent cells with age, and their initial benefit is then overtaken by detrimental functions exerted by chronic secretion of SASP factors and consequent tissue function dysfunctions, aberrant paracrine senescence, and chronic inflammation. In accordance to this notion, elimination of senescent cells (by using so‐called senotherapies) in aged and/or diseased conditions is sufficient to alleviate onset and progression of dysfunctions and pathology (Paez‐Ribes, 2019).

**Figure 1:**
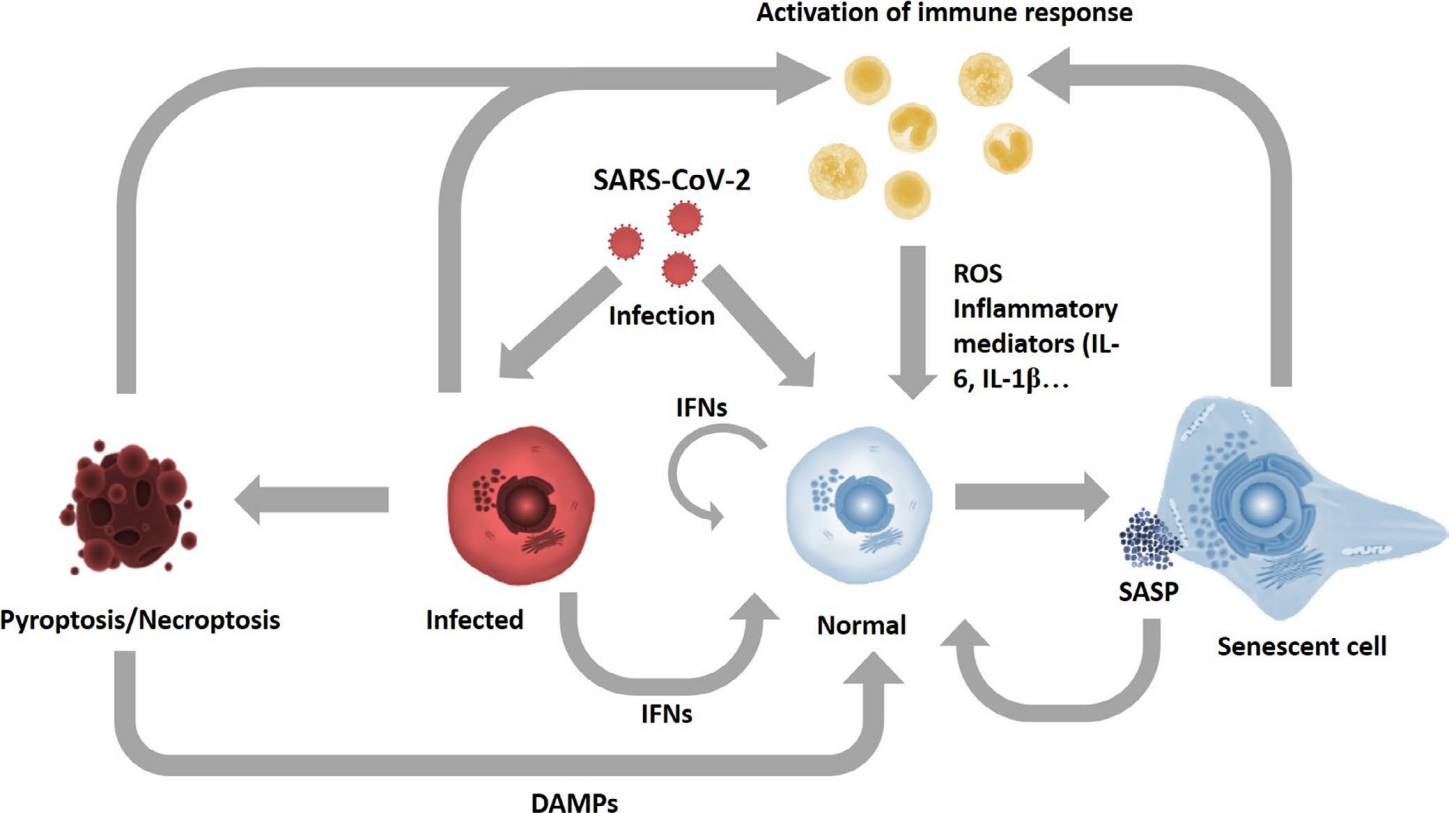
Potential pathways of senescence induction as a consequence of SARS‐CoV‐2 Infection. Viral infection can promote senescence induction directly or indirectly by increasing IFNs secretion from infected cells and DAMPs release from cells undergoing pyroptosis or necroptosis. Senescent cells can spread senescence in the environment via SASP factors. Additionally, anti‐viral mechanisms can activate the immune system which can further propagate senescence as a consequence of ROS and inflammatory mediators release

Interestingly, cellular senescence can be prematurely induced by viral infections via cell and/or non‐cell autonomous mechanisms. Some viruses can cause DNA damage (Martinez, 2016) or cell fusion (Chuprin, 2013) leading to a senescence state. In addition, infected cells activate anti‐viral responses which include the release of type I and III interferons (IFNs) and other pro‐inflammatory mediators (Newton et al., 2016) upon activation of pattern recognition receptors (PRRs) by pathogen‐associated molecular patterns (PAMPs). Interestingly, prolonged exposure to IFN‐γ and IL‐6 has been shown to induce senescence in normal cells, suggesting that infected, but not necessarily senescent, cells might activate a senescence in the surrounding environment (Kandhaya‐Pillai et al., 2017; Kim et al., 2009). Furthermore, the anti‐viral cyclic GMP‐AMP synthase (cGAS)‐stimulator of interferon genes (STING) signaling pathway has been recently shown to regulate senescence phenotypes (Dou et al., 2017; Hari et al., 2019). Beside these data, the fact that many viruses evolved proteins that can prevent infected cells from undergoing senescence (Reddel et al., 2010), and senescent cells are resistant to some viral infections (Baz‐Martinez et al., 2016) suggest that cellular senescence might act as an anti‐viral defense mechanism and that the number of senescent cells is likely to be elevated in infected individuals (Figure 1).

SARS‐CoV and SARS‐CoV‐2 trigger a “cytokine storm,” the release of an array of inflammatory cytokines and chemokines such as CXCL‐10, CCL‐2, IL‐6, IL‐8, IL‐12, IL‐1β, IFN‐γ, and TNF‐α. Many of these factors have the potential to induce “paracrine” senescence via prolonged cytokine signaling (Acosta et al., 2013; Kandhaya‐Pillai et al., 2017; Kim et al., 2009) (Figure 1). In addition, viral infections might propagate senescence via necroptosis and pyroptosis (Tang et al., 2019), two types of inflammatory cell death mechanisms that release danger‐associated molecular patterns (DAMPs). SARS‐CoV can activate NLRP3 inflammasome (Chen et al., 2019), a main inducer of pyroptotic cell death, and SARS‐CoV‐2 is thought to induce pyroptosis (Yang et al., 2020). DAMPs bind to PRRs and non‐ PRRs DAMP receptors and activate the interferon response (Gong et al., 2020), which can further propagate senescence (Figure 1) (Kim et al., 2009; Schuliga et al., 2020).

In summary, older COVID‐19 patients are more likely to accumulate high levels of cellular senescence for three reasons: (a) the number of senescent cells is already elevated at the time of infection, and this can increase the possibility of paracrine senescence events; (b) older cells are more susceptible to enter senescence because of their decreased capacity to repair damage. In addition, aged tissues are less capable to eliminate senescent cells due to, at least in part, a decline in immune functions (see next section).

## 2 ASSOCIATION OF CELLULAR SENESCENCE WITH IMMUNE IMBALANCE, CHRONIC INFLAMMATION, AND EXCESSIVE IMMUNE RESPONSES

Diminished immune responses that accompany aging, a phenotype defined immunosenescence, can predispose the elderly to complications caused by viral infection (Keilich et al., 2019). Immunosenescence is a multifaceted phenomenon associated to a basal level of chronic inflammation and less effective anti‐viral responses. Phenotypically, immunosenescence manifests as loss of naive T cells and of effector cell functionality, decreased lymphocyte proliferation, increased number of terminally differentiated memory lymphocytes and of neutrophils, and dysregulated cytokines production. A significant correlation between age and SARS‐CoV‐2 viral load has been reported (Liu et al., 2020; To et al., 2020), and the presence of an immunosenescent phenotype, demonstrated by elevated neutrophils‐to‐lymphocytes ratio (NLR) (Qin et al., 2020) and high IL‐6 production (Ulhaq & Soraya, 2020), has been correlated to disease severity. Changes observed in immunosenescence can be attributed to various cell intrinsic and extrinsic (environmental) factors, and cellular senescence is heavily implicated in influencing these factors.

Intrinsic defects related to senescence have been documented in adaptive immune cells with age. CD3+ T cells including the CD4/CD8 fraction can interact with other immune cells to help stimulate antibody response to infection and present an increase in p16INK4A expression exponentially with chronological age and that was associated with an increase in plasma interleukin‐6 concentration (Liu et al., 2009). In mice, T cells that exhibit senescence‐like features were shown to increase with age (Tahir et al., 2015). Interestingly, induction of mitochondrial dysfunction, a prominent aspect affecting many old tissues, in T cells promotes the acquisition of a senescence‐like phenotype (Baixauli et al., 2015). Using the same model, a recent study has shown that T cells with dysfunctional mitochondria have a reduced capacity to clear viral infections and strikingly, contributed to the accumulation of circulating cytokines termed “type‐1 cytokine storm” that act as a systematic inducer of senescence in several tissues (Desdin‐Mico et al., 2020). It is interesting to note that patients with COVID‐19 pneumonia have an increase in the proportion of T cells that display exhaustion/senescence markers (De Biasi et al., 2020). In addition to senescent‐like T cells, senescent‐like B lymphocytes also express inflammatory SASP factors that accumulate with age (Frasca et al., 2017) and IL‐6 and TNF‐α have been shown to induce premature senescence in non‐senescent T cells in a paracrine manner and are also increased in patients with severe COVID‐19 (Liu et al., 2009; Ulhaq & Soraya, 2020). Together, these results suggest potential implications in humans for senescence‐associated immune dysfunctions, with particular relevance to viral infections that partly rely on cellular immunity such as in the case of SARS‐CoV‐2.

Immunosurveillance by the innate immune system is the first responder to viral infections and other stresses. NKG2D ligands are surface proteins upregulated on viral‐infected cells that act as key activators of NK cells and facilitate immune clearance (Lanier et al., 2015). It has been reported that in different models of stress‐induced senescence in human fibroblast, including lung fibroblasts, and in some epithelial and tumor cells, NKG2D ligands are upregulated. However, a subset of senescent cells can evade NK cell immune detection by shedding NKG2D ligands through autocrine and paracrine MMP‐mediated proteolytic cleavage (Munoz et al., 2019; Sagiv et al., 2016). Since SASP MMPs are diffusible and senescent cells generally accumulate with age, early NK‐mediated immune response to SARS‐CoV‐2 in the elderly may be blunted by a senescence‐associated and MMP‐mediated NKG2D ligand shedding from infected cells, leading to impaired SARS‐CoV‐2 clearance.

Dendritic cells (DCs) have an important role in bridging innate and adaptive immunity. In the lungs, DCs reside in an “immature” state until they recognize and capture inhaled materials. Upon capturing antigens, in addition to the presence of proper stimulation, DCs migrate to draining regional lymphoid tissues, where they play a crucial role for activation of naïve T cells (Condon et al., 2011). The ability of DCs to migrate from the site of infection to the lymph nodes in response to many respiratory viral infections, including SARS‐CoV, is considerably impaired with increasing age, resulting in diminished T‐cell responses (Zhao et al., 2011). Interestingly, impaired DC migration has been shown to be mediated, in part, by increased levels of the lipid mediator prostaglandin D2 (PGD2) in the respiratory tract with age (Zhao et al., 2011), and prostaglandins secretion is significantly increased in senescent lung cells (Wiley et al., 2019; Zdanov et al., 2007). Thus, it is possible that age‐associated accumulation of senescent cells is responsible for the increased PGD2 levels in the respiratory tract.

The thymus is the primary central T lymphoid organ responsible for the development of naïve T cells and the establishment of immune tolerance. Thymic involution or regression is the most pronounced change in the aging immune system and seems to be an evolutionarily conserved process as it occurs in almost all vertebrates (Shanley et al., 2009). Thymic involution is associated with a progressive decline in the production of new T lymphocytes (Petrie et al., 2002) which eventually leads to reduced TCR repertoire diversity (Goronzy et al., 2007) and decreased capacity of mounting a sufficient adaptive immunity in response to infection (Palmer et al., 2018). Interestingly, cellular senescence has been linked to thymic involution both in mice and humans (Aw et al., 2008; Barbouti et al., 2019; Burnley et al., 2013), and could therefore be a likely contributor to age‐associated thymic inflammation and atrophy (Sempowski et al., 2000).

Lymph nodes (LNs) are a major site for the maintenance of naïve T cells (Brown & Turley, 2015). Upon aging, LNs show signs of morphological degeneration, changes in the histoarchitecture (including fibrosis), and numerical decline (Ahmadi et al., 2013; Hadamitzky, 2010). Subsequently, this altered tissue environment fails to support naïve T‐cell homeostasis (Becklund et al., 2016), contributes to suboptimal immune defense (Thompson et al., 2019), and compromises the priming of early adaptive immune responses upon viral infection (Richner et al., 2015). LNs stroma is populated with different subset of cells that are crucial for immune regulation (Chang & Turley, 2015). Some of these cells were shown to have an age‐related reduction in proliferation upon viral infection, potentially correlated to increased senescence (Masters et al., 2019).

The spleen is another secondary lymphoid organ that is affected by age. As is the case of LNs, the spleen also shows microarchitecture abnormalities (Turner & Mabbott, 2017) and different publications have demonstrated that senescence‐associated markers accumulate in the aging spleen (Baker et al., 2016; Wang et al., 2009) with splenic stromal cells expressing high IL‐6 levels (Park et al., 2014). Interestingly, clearing senescent cells from irradiated spleen can improve T‐cell and macrophage functions, suggesting a role of senescent cells in promoting immune dysfunctions (Palacio et al., 2019). In accordance, different SASP factors can affect the response of different immune cells: TNF‐α can accelerate T cells' replicative senescence (Parish et al., 2009) and decrease activation and class switching in B cells (Frasca et al., 2012), while CCL‐2 expressed by stromal cells can limit antibody‐forming cells (AFC) survival (Dasoveanu et al., 2020).

All of the mentioned studies implicate that cellular senescence is a likely contributor to the immunosenescence phenotype observed in aging organisms which could predispose COVID‐19 patients to develop severe‐to‐lethal pathogenesis.

## 3 CONTRIBUTION OF CELLULAR SENESCENCE TO TISSUE DAMAGE

### 3.1 Contribution of senescence to lung damage/failure

Once SARS‐CoV‐2 is inhaled, it is likely to first bind to ACE2 on epithelial cells in the nasal cavity and start replicating. Following this stage, the virus propagates in the respiratory tract and triggers a more robust immune response. Once reaching the gas exchange units of the lung, SARS‐CoV‐2 preferentially infects alveolar type II cells, the larger cells involved in surfactant secretion. About 20% of infected patients can develop pulmonary infiltrates, and some of these seem to progress to severe disease (Mason et al., 2020; Zhao et al., 2020). Main lung pathological features of COVID‐19 patients resemble those seen in SARS and Middle Eastern Respiratory Syndrome (MERS): pulmonary edema, microthrombi, diffuse alveolar damage with hyaline membrane formation (Carsana et al., 2020; Fox et al., 2020), and evidence of pneumocyte desquamation suggesting the presence or progression to acute respiratory distress syndrome (ARDS) (Xu et al., 2020). Importantly, an aberrant healing response can promote excessive tissue fibrosis, impair regeneration and remodeling, and eventually lead to suboptimal recovery (Mason et al., 2020).

Lungs, like many other organs, show a progressive decline in function with increasing age as a result of structural and physiological changes (Miller et al., 2010). Because the lung is constantly exposed to external environmental stressors, it is conceivable that lung aging results from a combination of external and internal factors that increases the risk of chronic lung diseases (Meiners et al., 2015). Notably, the incidence of degenerative chronic lung diseases, namely chronic obstructive pulmonary disease (COPD) and idiopathic pulmonary fibrosis (IPF), dramatically rises with age (Meiners et al., 2015), and unsurprisingly the elderly have higher morbidity and mortality after acute lung injury relative to younger adults (Rubenfeld et al., 2005). Different studies have shown that aged lungs show uncontrolled inflammatory responses in murine models of acute lung injury, including models of viral infection (Kling et al., 2017; Yin et al., 2014). In addition to imbalanced immune responses, defects in tissue maintenance and repair can also play a role in the susceptibility to lung damage. It is known that COPD and IPF increase the vulnerability to pneumonia (Raghu et al., 2011; Torres et al., 2013). Importantly, patients with lung COPD and IPF show an increase in senescence‐associated markers, and the removal of senescent cells alleviate progression of these pathologies in preclinical mouse models (Parikh et al., 2019). The contribution of cellular senescence to lung dysfunction occurs via different cell and non‐cell autonomous mechanisms. Senescent stem/progenitor cells reduce lung regenerative capacity, a major age‐associated hallmark in the lungs (Hamsanathan et al., 2019; Kathiriya et al., 2020). SASP factors IL‐6, IL‐8, IL‐1, and TNF‐α might be crucial for chronic inflammation (Kumar et al., 2014; Tsuji et al., 2010), while TGF‐β, PAI‐1, and MMPs might contribute to fibrosis (Lomas et al., 2012) and stimulate the expression of fibrotic genes, such as ACTA2, COL1A1, COL1A2, and FN1, in surrounding non‐senescent cells (Schafer et al., 2017).

### 3.2 Contribution of senescence to multi‐morbidity

COVID‐19 is often a multi‐organ disease. SARS‐CoV‐2 replication occurs in tissues outwit the lung, including the intestinal and renal epithelium (Chu et al., 2020) and is well recognized to be associated with clinical manifestations including but not limited to, diarrhea, derange liver function tests, kidney and brain dysfunction. Notably, symptom complexes dominated by joint and muscle pain or gastrointestinal disturbance rather than respiratory symptoms (such as cough) have been described (Docherty et al., 2020). In addition to impaired oxygenation due to lung syndromes, multi‐organ failure is usually observed in severe COVID‐19 cases (Guan et al., 2020), particularly those in Intensive Care Units.

The direct cause of multi‐organ failure in COVID‐19, as in other critical illnesses, is not clear but the major contributors are likely to be the direct viral infection, and an excessive immune response, mainly as a result of the cytokine storm (Li et al., 2020).

Systemically, microvasculature dysfunction is common in infected patients (Varga et al., 2020), with abnormal coagulation and thrombosis observed at late disease stages and associated to poor prognosis (Tang et al., 2020). Aberrant coagulation is due to endothelial activation and damage but can lead to damage in multiple tissues, increase cardiovascular events, and cause pulmonary embolism (Connors & Levy, 2020; Escher et al., 2020). Identified risk factors for vascular dysfunction are aging, obesity, diabetes, and hypertension—conditions associated to severe reactions to SARS‐CoV‐2 infection and by high number of senescent cells. Accumulating evidences suggest that cellular senescence plays an important role in vascular dysfunction. As mentioned before, SASP factors such as IL‐6 can help amplify SARS‐CoV‐2—driven inflammation. It is interesting to note that soluble IL‐6 receptor (sIL‐6R) is part of the SASP (Garbers et al., 2013). sIL‐6R activates IL‐6 signaling in cells that do not express membrane‐bound IL‐6 receptor (mIL‐6R), such as endothelial cells. This can lead to the activation of IL‐6 signaling in different types of cells, with consequent exacerbation of the inflammatory response, but also to senescence propagation (Kojima et al., 2013). Along with inflammatory factors, it has been shown that senescent cells secret different hemostasis‐related factors that favor clotting (Wiley et al., 2019). Endothelial senescent cells can promote a prothrombotic state via upregulation of factors that induce platelet aggregation (e.g., PAI‐1, thromboxane A2, and vWF), and downregulation of factors that inhibit aggregation (e.g., eNOS, prostacyclin, and thrombomodulin) (Bochenek et al., 2016). Thus, cellular senescence might elevate the risk for thrombosis by shifting the balance between anti‐ and procoagulant pathways. Furthermore, senescent endothelial cells have an impaired barrier integrity and reduced tight junctions (Krouwer et al., 2012; Yamazaki et al., 2016). Vascular regeneration might be an essential factor in recovery and prevention of damage progression in response to infection. Cellular senescence can alter the repair capacity of the endothelium and decrease angiogenesis through the impairment of proliferation and function of endothelial progenitor cells thus leading to persistent damage (Ungvari et al., 2018). Cellular senescence is also linked to pulmonary hypertension (Feen et al., 2019), which could potentially restrict blood flow to the lungs and induce capillary damage eventually leading to hypoxemia and ARDS. Interestingly, endothelial cells might be induced to senescence by SARS‐CoV‐2 infection. The binding of SARS‐CoV‐2 to ACE2 receptor and subsequent entry can lead to the suppression of the activity of this enzyme. This can result in decreased Ang‐(1–7) generation, and consequently increased Ang II levels (South et al., 2020). Increased activity of ACE–Ang II axis relative to that of ACE2–Ang‐(1–7) axis might help driving cellular senescence, especially in the vascular system (Mogi et al., 2020). Taken together, high level of vasculature senescence, either already present at time of infection or induced by the reaction to SARS‐CoV‐2, can contribute to the exaggerate vascular dysfunction observed in severe cases of COVID‐19.

Myocardial dysfunction is common in COVID‐19 with the cardiac myocytes being involved in the infectious process in SARS (Oudit et al., 2009) and SARS‐CoV‐2 (Wang et al., 2020). Cardiac troponins, a marker of cardiac myocyte damage are frequently elevated in COVID‐19 and are a marker of poor prognosis (Guo et al., 2020). Cellular stress in cardiac myocytes has been associated with a raft of molecular markers of senescence (Kajstura et al., 2000; Sheydina et al., 2011) and are themselves associated with impaired cardiac function. Acute cardiac damage promotes mesenchymal senescence and fibrosis (Zhu et al., 2013). Similarly, chronic cardiac disease may have associations with the establishment of senescence in the heart (Shimizu & Minamino, 2019), particular of cardiac fibroblasts. Functional studies depleting senescent cells have linked the senescence to perpetuation of atherosclerosis and cardiac fibrosis formation (Childs et al., 2018).

Vascular complications in late disease may promote or worsen damage to other organs. However, it is notable that some organs which may not be directly infected by the virus are often affected early during the disease. For example, abnormal liver function tests are present in up to three quarters of patients with COVID‐19 infection with their elevation being associated with poor patient outcomes (Cai et al., 2020). These abnormalities are typically elevated levels of hepatocyte‐specific transaminases (ALT and AST). Hepatocytes, unlike other cells in the liver and the bile ducts, are not themselves directly tropic to the virus, so their damage appears to be an indirect event. While vascular phenomena in COVID‐19 may contribute to this (McGonagle et al., 2020), deranged liver function tests may be seen in other forms of systemic illness, including sepsis, and may affect liver function via hepatocyte damage and indirect tissue injury (Roelofsen et al., 1994; Yan et al., 2014). Acute or chronic tissue damage in hepatocytes is sufficient to induce a senescence‐like state (Aravinthan & Alexander, 2016; Bird et al., 2018). The liver was the first organ in which senescence was shown to be capable of paracrine spread between tissue epithelium in vivo, both between hepatocytes (Bird et al., 2018) and between the biliary tree and hepatocytes (Ferreira‐Gonzalez et al., 2018). Whether cellular senescence may be spread in an endocrine manner during COVID‐19, both within and between tissues, remains to be established. Similarly, if the presence of a underlying senescence burden in tissues predisposes to the establishment of the sort of positive feedback that drives senescence spread within the liver through TGF‐β (Bird et al., 2018) is worthy of further investigation.

Elderly patients with severe cases of COVID‐19 are also more likely to experience a host of neurological symptoms, suggestive of central nervous system infection, including impaired consciousness and loss of smell (Mao et al., 2020; Yan et al., 2020). During the 2003 SARS outbreak evidence of neural tissue infection was found (Hamming et al., 2004; Xu et al., 2005; Zhou et al., 2020). A recent report has found evidence of the SARS‐CoV‐2 virus also appearing in cerebrospinal fluid, but how the virus enters this protected tissue is currently unknown (Wu et al., 2020a). Vascular cells of the blood‐brain barrier that help isolate the CNS from infection can become senescent and contribute to functional decline of the structure with age (Yamazaki et al., 2016). Interestingly, some patients showed neurological symptoms before pulmonary features, suggesting the possibility of infection via an alternative route involving the nervous tissue (Mao et al., 2020). Progression of age‐associated senescence in the blood‐brain barrier may therefore be an important factor in CNS infection and disease severity in elderly COVID 19 patients.

It is essential to note that, depending on the severity of the disease, recovered patients might develop long‐term health consequences (Chiumello et al., 2016; Inciardi et al., 2020; Wu et al., 2020b). Previous SARS‐CoV and MERS‐CoV epidemics were both associated with significant incidence of long‐term fibrotic lung disease as well as involvement of other organs (Das et al., 2017; Ong et al., 2004; Zhang et al., 2020). Early reports have suggested evidence of impaired lung function in patients recovering from SARS‐CoV‐2. As it is presented, SARS‐CoV‐2 infection has the potential to promote senescence spread across different tissues in multiple ways. Accumulation of senescent cells after infection might predispose individuals to premature aging in different organs, contributing to long‐term consequences and accelerating numerous age‐related pathologies. Therefore, evaluating the presence and role of senescence caused by viral infection might represent an important predictor for the consequences of long‐term damage.

## 4 CONTRIBUTION OF CELLULAR SENESCENCE TO VACCINATION

Reduced vaccine efficacy in the elderly is a concern as COVID‐19 vaccines enter clinical trials. Evidence is mounting that both humoral and cellular immunity is critical in battling COVID‐19 (Tay et al., 2020). Bridging the gap between cellular senescence and adaptive immunity are a group of stress‐inducible proteins called sestrins that play a direct regulatory role in T cells that have acquired senescent‐like features. Sestrin expression in senescent‐like CD4+ T cells impaired AMPK signaling and T‐cell function through a novel ERK/JNK/p38 MAPK activation pathway. Sestrin knockdown partially reversed the senescent‐like phenotype and enhanced T‐cell responsiveness in vitro, while Sestrin knock‐out mice were able to produce higher antibody titer against influenza infection, demonstrating a critical function in overall immune response (Lanna et al., 2017). Similarly, expression of Sestrin impairs CD8+ senescent‐like T cells activity by blocking TCR signaling and shifting effector activity toward an NK cell‐like phenotype. Conversely, down‐regulating Sestrin expression in these cells resulted in partial recovery of T‐cell function and coincided with the disappearance of NK cell ligand receptors NKG2A and NKG2D. In this way, cellular senescence might directly drive a shift away from adaptive immunity toward innate immune responses in the elderly with important implications for vaccine development (Pereira et al., 2020). Senescent‐like B cells expressing p16 and constitutively secrete a SASP‐like profile including TNF‐α and IL‐6 also appear in older organisms (Frasca et al., 2017). Experiments show that TNF‐α reduces B‐cell class switching and hypermutation required for high‐affinity antibody generation and that blocking TNF‐α with antibodies improved B‐cell activation (Frasca et al., 2014; Signer et al., 2008). Autocrine and Paracrine effects of TNF‐α signaling among circulating B cells suggests that as senescent‐like B cells accumulate, the overall population effector function will be diminished. Even more fundamentally, the spatiotemporal regulation of B‐cell development in the bone marrow arises from a complicated interplay of chemokine and cytokine gradients between bone marrow cellular niches. CXCL‐12 is a chemokine that participates in this gradient and directs developing B‐cell migration (Tokoyoda et al., 2004). Expression of CXCL‐12 can be seen in senescent stromal cells cultured from old mice and is ameliorated with ABT263 (Kim et al., 2017). This aberrant regulation of CXCL‐12 may degrade the optimal cellular niche gradient, affecting B‐cell fate and function by reducing compartmentalization of early precursor B cells, and interfering with B‐cell migration from the bone marrow.

DNA‐based vaccines for use against COVID‐19 are already entering clinical trials (ClinicalTrials.gov Identifier: NCT04336410). Compared to traditional vaccines, they have the advantage of being quick to develop and rapidly scalable at cost. These vaccines partially depend on antigen presentation on MHC class I molecules by somatic cells transfected with DNA vectors encoding viral proteins. CD8+ T‐cell detection of expressed viral antigens is an important element of DNA‐vaccine efficacy, as well as innate NK cell activity (Ahmad et al., 2012; Zhu et al., 2011). DNA‐Vaccines are administered to skin or muscle tissue, both of which display an accumulation of senescent cells with age (Waaijer et al., 2012; Wang et al., 2009) that may interfere with vaccine efficacy. In dermal tissue, senescent skin cells display upregulation of surface HLA‐E proteins that inhibit co‐stimulatory NKG2A receptors on both NK cells and CD8+ T cells, impeding immune cell activation (Pereira et al., 2019). Additionally, immune cell‐stimulating NKG2D ligands present on transfected cells are suppressed in senescent cells by autocrine MMP proteolytic cleavage (Lanier et al., 2015; Munoz et al., 2019). Furthermore, MMPs are part of the SASP and may diffuse into surrounding tissue further dampening NKG2D signaling. DNA‐vaccine immunogenicity also relies on a STING‐driven type I IFN induction pathway and activation of innate and CD8+ T cells (Suschak et al., 2016). As senescent cells already display constitutive STING activation (Dou et al., 2017) while evading immune detection, it is likely that DNA‐vaccination will generate only marginal immunogenicity in these cells by this mechanism. Senescent cells may therefore act as DNA vector decoys in the elderly; and dampen antigen‐specific immune response.

## 5 FUTURE OUTLOOK: CELLULAR SENESCENCE AS BIOMARKER AND TARGET FOR COVID‐19

**Figure 2:**
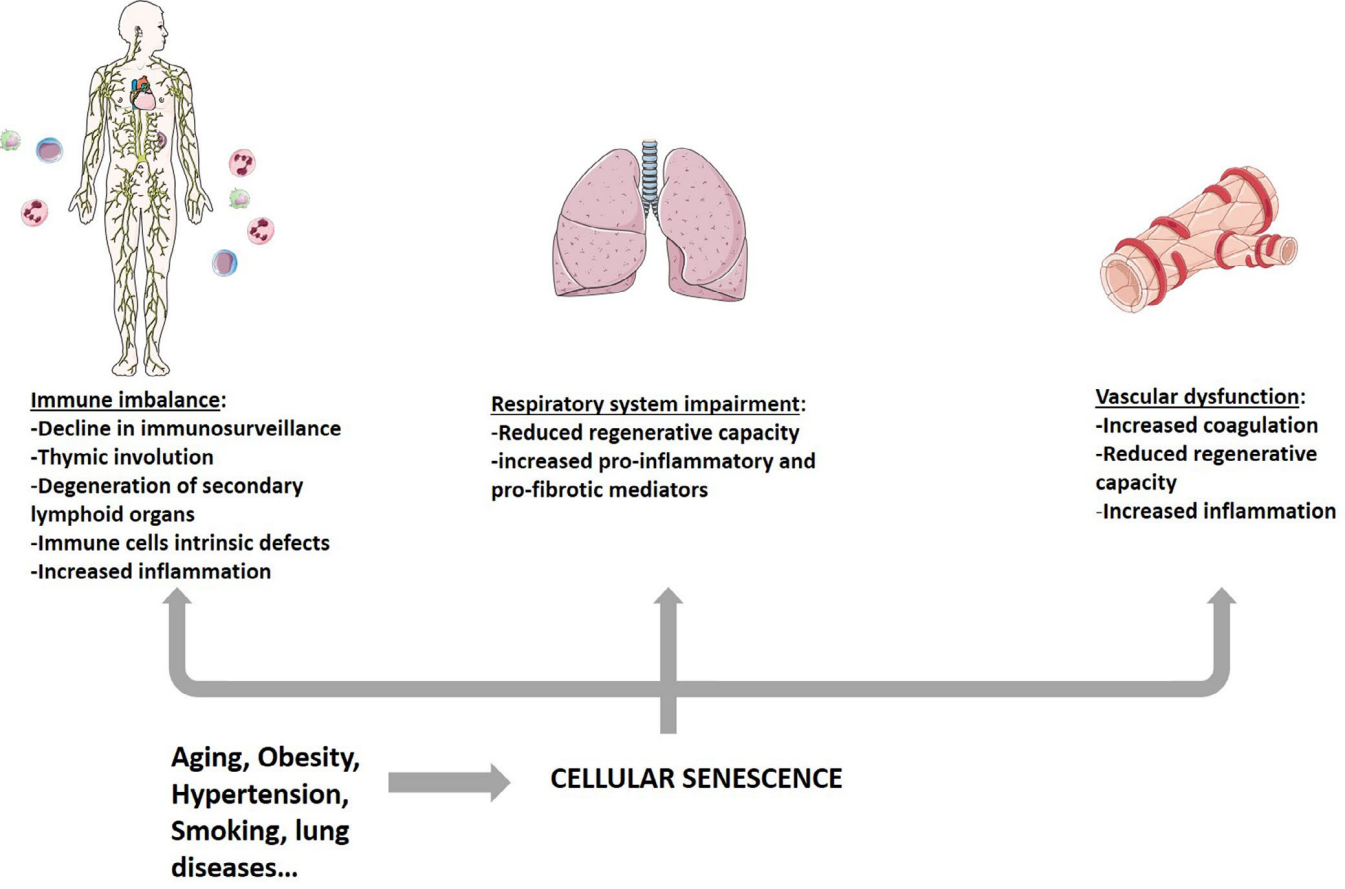
Effects of cellular senescence on different body systems might increase the risk for developing severe COVID‐19. Various factors can induce multi‐tissue accumulation of senescent cells. Cellular senescence can lead to immune imbalance, including weak adaptive immunity and exaggerated inflammatory response. In lungs, senescence can decrease regenerative capacity and enhance aberrant healing response and tissue fibrosis. Vascular function is also known to be highly affected by cellular senescence through different mechanisms including increased thrombotic and inflammatory responses and decreased regeneration

High burst of senescence is normally observed in older individuals, which are by far the most likely to develop severe‐to‐lethal COVID‐19, and can be caused by direct and indirect virus‐mediated mechanisms (Figure 1). Senescent cells might contribute to severe reactions to SARS‐CoV‐2 by provoking immune imbalance and by mediating local and distant damage (Figure 2). Thus, measuring the abundance of senescent cells can serve as a biomarker to predict for COVID‐19 severity, while targeting senescence‐associated mechanisms prior and after SARS‐CoV‐2 infection has the potential to limit severe and even lethal damages. Moreover, reduction of senescence might also improve the efficacy of vaccinations.

Measuring senescence in human tissues remains a challenging task. In recent years, it has become evident that the senescence phenotype is highly heterogenous, and neither a specific nor a universal senescent marker exists (Hernandez‐Segura et al., 2018). However, tissue levels of the CDK4/6 inhibitor p16INK4A or of the lysosomal senescence‐associated β‐galactosidase could be used as a surrogate tool for measuring senescence (Gorgoulis et al., 2019).

Because of the recognized detrimental effects of senescent cells in various age‐related disorders, there has been an exponential interest in the development of senotherapies. Senotherapies are based on compounds able to either eliminate senescent cells (senolytics) or interfere with aspects of senescence such as the SASP (senostatics). Senotherapies have recently entered clinical trials, and two strategies—the combination of the protein kinase inhibitor Dasatinib with the flavonoid Quercetin (D + Q) and UBX0101, a compound disrupting the interaction between Mdm2 and p53—have shown tolerability and a safe profile. Interestingly, compounds with predicted senolytic or senostatic properties are currently in clinical trials for COVID‐19 (Table 1): quercetin, shown to be senolytic in various preclinical models (Russo et al., 2020), is tested as prophylactic treatment; anakinra, tocilizumab, pirfenidone, and ruxolitinib, all shown to have the capacity to inhibit parts of the SASP (Hubackova et al., 2012; Laberge et al., 2015; Orjalo et al., 2009; Rodier et al., 2009; Soto‐Gamez & Demaria, 2017), are used to treat the hyperinflammatory syndrome associated with severe COVID‐19 disease; sirolimus, an mTOR inhibitor which can reduce SASP and prevent senescence induction (geroconversion) (Blagosklonny et al., 2018; Laberge et al., 2015), is tested as treatment against SARS‐CoV‐2 spread and it is thought to prevent severe progression in COVID‐19 (Omarjee et al., 2020); metformin, an anti‐diabetic drug that activates AMPK and lower NFkB leading to SASP reduction (Moiseeva et al., 2013), is tested as an intervention against the acute respiratory syndrome.

**Table 1:** List of selected COVID‐19 trials using potential senotherapeutics

Studies^a^	Drug	Enrollment^a^	Senescence‐related target	Study identifier
110/51	*Tocilizumab* *Sarilumab* b	*N* = ≤5000	IL6‐Receptor/SASP inhibitor	IRCT20151227025726N13 NCT04315298
4	*Pirfenidone*	*N* = ≥500	TGF‐β, TNF‐α/SASP inhibitor	NCT04282902 CHICTR2000030333 CHICTR2000030892 CHICTR2000031138
4	*Anakinra*	*N* = ≥100	IL1‐Receptor/SASP inhibitor	NCT04364009 NCT04341584 NCT04341584 NCT04318366
2	*Ruxolitinib*	*N* = ≥50	JAK/STAT inhibitor	IRCT20160310026998N11 IRCT20131129015584N2
2	*Sirolimus* b	*N* = ≥50	mTOR/SASP inhibitor	NCT04371640 NCT04341675
1	*Metformin*	*N* = 200	AMPK activators/SASP inhibitor	IRCT20160310026998N11
1	*Quercetin* b	*N* = 50	PI3K/AKT/P53/Serpine/ HIF 1 α	NCT04377789

a Data collection: https://www.covid​-trials.org/.

b Not completed studies.

Based on these concepts, evaluation of senescence markers should be considered in treated and non‐treated COVID‐19 patients and at different time points during and after SARS‐CoV‐2 infection. This information can shed light on the potential to use the burst of cellular senescence as a biomarker to predict disease severity, but also as a pharmacological target to reduce the short‐ and long‐term consequences of the disease.

## CONFLICTS OF INTEREST

M.D. is co‐founder, shareholder, and advisor of Cleara Biotech.

## AUTHOR CONTRIBUTIONS

J.N. and M.D. wrote the manuscript with input from M.B., S.M., and T.G.B. M.D. supervised and planned the writing of the manuscript.
